# Coracoid Process Morphology using 3D-CT Imaging in a Malaysian Population

**DOI:** 10.5704/MOJ.1707.012

**Published:** 2017-07

**Authors:** II Imma, NM Nizlan, AR Ezamin, S Yusoff, MH Shukur

**Affiliations:** Department of Orthopaedics, Universiti Putra Malaysia, Serdang, Malaysia; ^*^Department of Orthopaedics and Traumatology, Universiti Kebangsaan Malaysia, Cheras, Malaysia

**Keywords:** coracoid process, coracoid anatomy, coracoid dimension, Malaysian population

## Abstract

**Introduction:** The aims of this study are to define the coracoid process anatomy in a Malaysian population, carried out on patients in Hospital Serdang with specific emphasis on the dimension of the base of coracoid process which is important in coraco-acromial (CC) ligament reconstruction, to define the average amount of bone available for use in coracoid transfer, and to compare the size of coracoid process based on gender and race, and with findings in previous studies.

**Materials and Methods:** Fifteen pairs of computed tomography (CT) based 3-dimensional models of shoulders of patients aged between 20 to 60 years old were examined. The mean dimensions of coracoid were measured and compared with regards to gender and race. The data were also compared to previously published studies.

**Results:** The mean length of the coracoid process was 37.94 ± 4.30 mm. Male subjects were found to have larger-sized coracoids in all dimensions as compared to female subjects. The mean tip of coracoid dimension overall was 19.99 + 1.93mm length × 10.03 + 1.48mm height × 11.63 + 2.12mm width. The mean base of coracoid dimension was 18.96 + 3.71mm length × 13.84 + 1.76mm width. No significant differences were observed with regards to racial denomination. The overall coracoid size measurements were found to be smaller compared to previous studies done on the Western population.

**Conclusion:** This study may suggest that Malaysians have smaller coracoid dimension compared to Caucasians. The findings further suggest that the incidence of coracoid fracture and implants pull out in Malaysian subjects may be higher.

## Introduction

The coracoid process is significant as a bony landmark in many surgical procedures around the shoulder joint. The coracoid is used by surgeons for graft in coracoid transfer procedure for shoulder instability and also for coracoacromial (CC) ligament reconstruction procedures^1–3^. The anatomical dimension of coracoid therefore is crucial as the size will determine how much of the coracoid process can be harvested as a graft during coracoid transfer procedures. In CC ligament reconstruction, inappropriate size of drill, size of implants and even the inaccurate trajectory of tunnel drilling can increase the risk of coracoid fracture and implant pull-out. By having our own data for the population in this region, it may provide information to surgeons on how much cortical wall remains during tunnel preparation in CC ligaments reconstruction.

Various studies have been carried out on coracoid morphology using dry bone, fresh cadaveric bone and computed tomography. However, most do not provide comprehensive reports of the entire coracoid anatomical dimensions. Instead, the coracoid process was studied partly following the procedure they were discussing. For example, the entire length of coracoid process and its tip were measured for coracoid transfer procedure for gleno-humeral instability and its base was most discussed in CC reconstruction surgery. There are no studies done in Malaysia thusfar and only limited literatures are available that defines the anatomical dimension of coracoid process using CT scan 3D reconstruction.

As Asian individuals are smaller in physical size compared to Caucasians, using surgical devices and implants that are designed for the Caucasians will impose inappropriate risks of intra-operative and post-operative coracoid fractures and implant pull-outs. Therefore, the primary aim of this study is to define the entire coracoid anatomy and its measurement in our population by using CT scan 3D reconstruction and comparing its value to other previous studies. It is our hope that this data will be useful in the future to provide a cause for the incidence of coracoid fractures and implant failures with the usage of implants of sizes currently available in the market.

## Materials and Methods

This is a descriptive and prospective study. Fifteen patients were included in this study, involving 15 pairs of shoulder joints. The range of the age was between 20 to 60 years. The procedure was performed in the Radiology Department, Hospital Serdang, Malaysia. All patients who were selected in this study were those with lung pathology that required CT scan of the thorax for diagnosis. Exclusion criteria were history of fracture involving the clavicle and coracoid process, arthritis around the shoulder joint, tumours around the shoulder and gleno-humeral and acromioclavicular joint instability

**Fig. 1:**
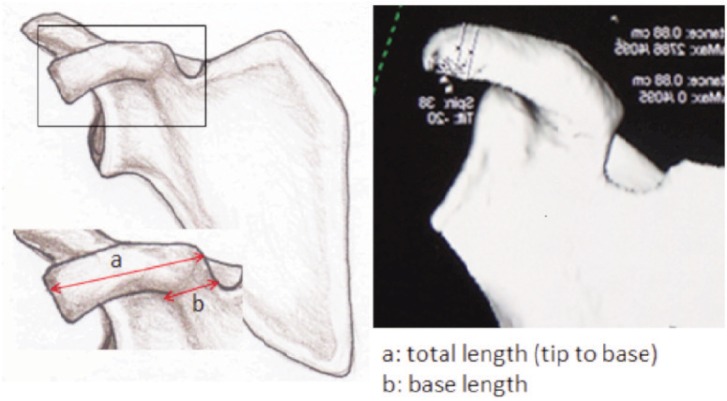
Coronal view of coracoid process.

**Fig. 2:**
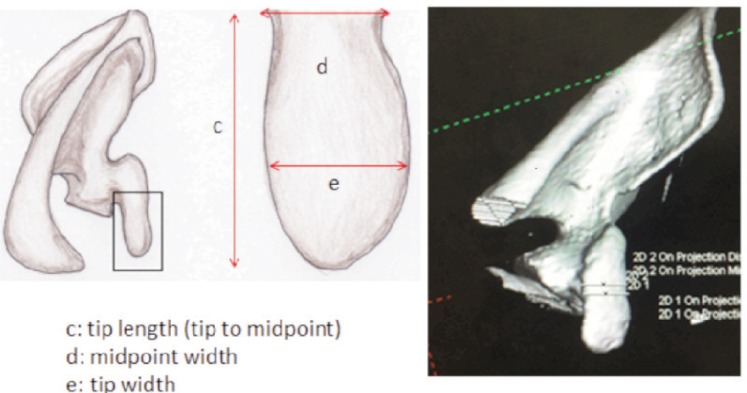
Axial view of coracoid process.

**Fig. 3:**
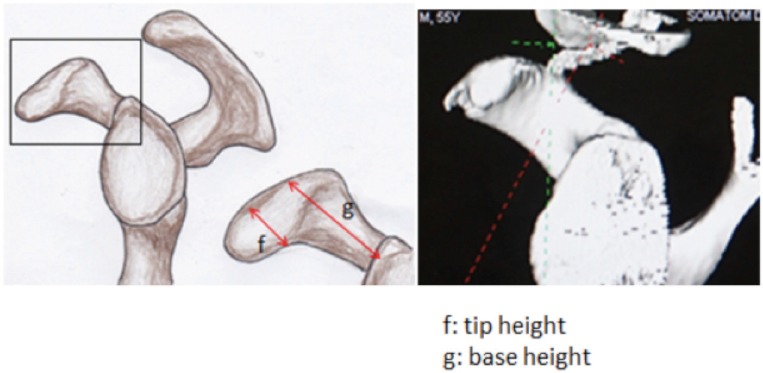
Sagittal view of coracoid.

All CT scans were performed on a 128/DE CT Scanner (Somatom Sensation Flash, Siemens Medical Solution manufactured in Erlangen, Germany). The scan covered an area from the upper thorax to the adrenal glands in craniocaudal orientation. The scanning protocol with a pitch of 1.0 acquisition of (128 × 2) × 0.6 mm (z-flying focus spot) and rotation time of 0.28 seconds was used with Tube A-80 kV at 382mAs and Tube B-140 kV at 90mAs. Automated tube current modulation (CareDose 4D, Siemens Medical Solution) was applied in this protocol. Images were reconstructed at a 0.75mm slice thickness in MPR and maximum-intensity-projection images and volume rendering. Image analyses were done using syngo.via (Siemens Healthcare) 3D Maximum Intensity Projection (MIP) with direct body bone removal as this is more representative. Analysis using multiplanar reconstruction (MPR) and volume rendering was allowed. Images from 3D reconstructed CT scan of the shoulder joints were cropped and devoid of structures other than scapula based on study by Rios3 (Fig. 1-3). The measurement was done using an electronic calliper. This process was conducted by two different individuals at different time using the same final images. The dimension of the coracoid process was measured in millimetres (mm) as 1) total length: a 2) tip of coracoid: c × e × f and 3) base of coracoid: b × d × g and each parameter was recorded in a spread sheet.

## Results

Data collected were analysed using the SPSS version 19.0 software to generate descriptive and analytical statistics. This study involved 15 subjects with the majority of the cases being female (60.0%). Ethnic Malay subjects contributed 73.3% of the cases while Chinese and Indian subjects contributed 13.3% each.

The mean total length of coracoid from tip to base in this study was 37.94 + 4.30mm. Mean coracoid length in male subjects was 40.88 + 3.87mm while in females the coracoid length was 35.98 + 3.49mm with average 5mm difference between male and female subjects. Male subjects had significantly larger coracoid processes compared to female subjects (Table I).

**Table I:** Measurement of coracoid process in this study

**View**	**Mean±SD (mm)**
Anterior view	
Total length	37.94±4.30
Base length	18.96±3.71
Lateral view	
Tip height	9.24±1.16
Base height	15.03±3.65
Superior view	
Tip length	20.98±2.90
Tip width	11.63±2.12
Midpoint width	13.84±1.76

All values are expressed as mean±SD

The mean tip of coracoid dimension was 19.99 + 1.93mm length × 10.03 + 1.48mm height × 11.63 + 2.12mm width. Meanwhile, the mean base of coracoid dimension was 18.96 + 3.71mm length × 13.84 + 1.76mm width (Table II).

**Table II:** Comparison of coracoid process according to gender

**View**	**Gender**	
	**Male (n=6)**	**Female (n=9)**
	**Mean±SD**	**Mean±SD**
Anterior view		
Total length*	40.88±3.87	35.98±3.49
Base length*	21.62±3.48	17.19±2.77
Lateral view		
Tip height*	10.18±1.24	8.61±0.56
Base height	16.47±3.70	14.07±3.49
Superior view		
Tip length	22.06±3.09	20.27±2.59
Tip width*	13.34±2.34	10.49±0.89
Midpoint width	14.11±0.53	13.66±0.89

^*^Significant if p-Value < 0.05

It is difficult to compare the parameters used in this study with other studies as the others had their own way of measuring the portions (some did not disclose the process in detail). Some studies used cadaveric bone and some used CT scan. This mixture of studies is definitely not uniform in term of measurements. However, their definition of the parameters was close to ours. All the measurements were tabulated in Table II for comparison.

## Discussion

The anatomy of the coracoid process is complex (Table III). It presents a curved shape. The base of the coracoid or the inferior pillar (vertical portion of the coracoid) originates off the antero-superior aspect of the glenoid vault^1^. It is connected to the superior pillar (horizontal limb of the coracoid) at the coracoid ‘elbow’ or junction and traverses laterally and terminates at the tip of coracoid^1^. The coracoid assumes a biomechanical function as a lever through which the muscular action of the coracobrachialis and short head of biceps tendon (conjoint tendon) and the pectoralis minor muscles exert force on the glenoid^4^.

**Table III:** Previous studies on measurement of the coracoid process

**Study**		**Coracoid length (mm)**	**Coracoid tip width (mm)**	**Coracoid tip height (mm)**	**Coracoid base width (mm)**	**Coracoid base height (mm)**
Lo *et al* (2004)	Pooled	22.7	15.9	10.4		
Rios *et al*(2007)	Pooled	45.2 ± 4.1			24.9 ± 2.4	11.9 ± 1.8
	Males	46.3 ± 3.3			25.4 ± 2.0	12.2. ± 1.7
	Females	40.7 ± 4.3			23.0 ± 2.8	10.5 ± 1.6
Salzmann *et al*(2010)	Males	46.0 ± 1.9			16.7 ± 2.9	15.4 ± 1.3
	Females	42.0 ± 1.4			13.0 ± 1.7	13.6 ± 1.7
Dolan *et al* (2011)	Pooled	45.6 ± 4.2	18.3 ± 1.8	11.5 ± 0.9		
Armitage *et al* (2011)	Pooled		15.0	10.5		
Coale *et al* (2012)	Pooled	45.0 ± 3.8			27.9 ± 2.5	
	Males	45.7 ± 3.7				
	Females	41.5 ± 2.3				

Known as the safe-zone ‘lighthouse’ during surgery, this tiny portion of the scapula has been used by surgeons largely to treat acromio-clavicular (AC) joint dislocation, Bankart lesion in shoulder instability and many more shoulder conditions^2,3,5^. However, these surgeries may also contribute to fracture of the coracoid process and implant failure. Hence, the authors are of the opinion that the size of coracoid process is an important factor in these surgeries.

Lo *et al*^5^ in their cadaveric study measured the dimension of the tip of the coracoid process while evaluating the anatomic relationship of the coracoid to the neurovascular structures that are at risk of injury during arthroscopic coracoplasty. In this study, the mean dimensions of the coracoid tip were 15.9mm × 22.7mm × 10.4mm (width × length × height).

Rios *et al*^3^ described the anatomy of the coracoid base and clavicle from fresh cadavers and dry bone specimens as they studied in detail the anatomic origin of the CC ligaments on the distal clavicle. The mean length from the base of coracoid process to the tip was 45.2 ± 4.1mm. The width and the height of the base of the coracoid were 24.9 ± 2.4mm and 11.9 ± 1.8mm respectively. The mean difference in the length of the coracoid process in males (46.3 ± 3.3mm) and females (40.7 ± 4.3mm) was 5.6mm. The width of the coracoid base was 25.4 ± 2.0mm in males and 23 ± 2.8mm in females and the height of the coracoid base was 12.2. ± 1.7mm in males and 10.5 ± 1.6mm in female. There was no difference noted in the coracoid measurements when comparing the values between Caucasians and African-Americans.

Salzmann *et al*^2^ studied the dimension and orientation of the CC footprints with respect to bony landmarks on coracoid. The measurements were obtained from fresh frozen cadaveric human shoulder. The length of the males and females coracoid was 46 ± 1.9mm and 42 ± 1.4mm respectively. The width of the base of coracoid was 16.7 ± 2.9mm in males and 13 ± 1.7mm in females while the height of the coracoid base was 15.4 ± 1.3mm in males and 13.6 ± 1.7mm in females. There was no significant ethnic difference in this study.

Dolan *et al*^6^ measured the tip of the coracoid process while studying its soft tissue attachments for coracoid transfer such as in Latarjet and Bristow procedures. The maximum length of coracoid transfer was 28.5 ± 5.1mm, measured from the tip to the ‘elbow’ of the coracoid. The mean coracoid length was 45.6 ± 4.2mm while the mean coracoid tip width and height were 18.3 ± 1.8mm and 11.5 ± 0.9mm respectively. The midpoint of the coracoid was 22.8 ± 2.1mm from the base or tip. The mean of the midpoint width and height were 16.1 ± 2.3mm and 13.5 ± 1.6mm respectively.

The mean length of the tip of the coracoid process in the present study was 20.98 + 2.90mm. Male subjects had mean tip length of 22.06 + 3.09mm, while female subjects had 20.27 + 2.59mm. This means a minimum coracoid graft of 20 mm can be harvested for the coracoid transfer procedure in our population. Young *et al*^7^ suggested a coracoid graft greater than 25 mm can be used routinely in Latarjet procedure, while Dolan *et al*^6^ suggested the maximum of 28 mm (18.1 + 1.8 mm mean tip) of coracoid could be harvested for the same procedure.

There are few other studies measuring the coracoid process using CT scan. Armitage *et al*^8^ and Coale *et al*^9^ have conducted studies using CT scan on tip and base of coracoid process respectively. Their result showed significant larger coracoid process than we found in our study. Coale *et al* also found that 91.3% of the shoulders had medial cortical breach by creating a tunnel based on the anatomic footprints of the CC ligaments. However, if a transclavicular-transcoracoid tunnel was created a little further than the anatomic footprint, it resulted in mean remaining medial and lateral wall thickness before cortical breach of 7.3 ± 1.7mm and 7.0 ± 1.6mm respectively (coracoid base width of 28mm). The distance of this tunnel from the anatomic midpoint of CC ligament footprints was 9.9 ± 2.2mm, resulting in non-anatomic CC ligaments reconstruction.

In theory, using a drill bit size of 5.0mm to drill a coracoid tunnel in our sample (mean coracoid base length of 18.96mm) results in remaining wall thickness of 7mm each side provided the tunnel is drilled in the central position. Given a tight window, eccentric tunnel preparation will increase the risk of cortical breach and coracoid fracture.

## Limitation of Study

Defining the dimensions of coracoid process in CT scan is a difficult task due to its tortuous shape^8,9,11^. The main challenge in this study is to identify the bony landmarks and portions of the coracoid process on the CT scan images. The 3D images have to be flipped and turned until they assumed the best position for measurement, as close to examining the native or cadaveric samples. Hence, it may not be accurate to compare this study and others. To reduce error in this study, the measurement was done by two independent observers. The sample size is also small, comparing the measurements in three different ethnic groups.

## Conclusion

This study showed that the Malaysian population has smaller coracoid process. A maximum of 23mm coracoid osteotomy can be safely used in coracoid transfer procedures. The findings also suggest that the incidence of coracoid fracture and implants pull-out following CC ligaments reconstruction surgery may be linked to the utilization of equipment and implants that are manufactured based on the Caucasian-sized coracoids. We recommend further study on biomechanics and effects of different drilling sizes used for CC ligaments reconstruction procedures to prove this hypothesis.
